# On the functional organization and operational principles of the motor cortex

**DOI:** 10.3389/fncir.2013.00066

**Published:** 2013-04-18

**Authors:** Charles Capaday, Christian Ethier, Carl Van Vreeswijk, Warren G. Darling

**Affiliations:** ^1^Brain and Movement Laboratory, Section of Biomedical Engineering, Department of Electrical Engineering, Danish Technical UniversityLyngby, Denmark; ^2^Laboratoire de Neurophysique et Physiologie du Systeme Moteur, CNRS UMR 8119, Université Paris-DescartesParis, France; ^3^Department of Physiology, Northwestern UniversityChicago, IL, USA; ^4^Department of Health and Human Physiology, University of IowaIowa City, IA, USA

**Keywords:** motor cortex, cortical circuits, motor map, cortical connectivity, microstimulation, neural mechanisms of cortical activity spread, multi-unit recording arrays, balanced neural networks

## Abstract

Recent studies on the functional organization and operational principles of the motor cortex (MCx), taken together, strongly support the notion that the MCx controls the muscle synergies subserving movements in an integrated manner. For example, during pointing the shoulder, elbow and wrist muscles appear to be controlled as a coupled functional system, rather than singly and separately. The recurrent pattern of intrinsic synaptic connections between motor cortical points is likely part of the explanation for this operational principle. So too is the reduplicated, non-contiguous and intermingled representation of muscles in the MCx. A key question addressed in this article is whether the selection of movement related muscle synergies is a dynamic process involving the moment to moment functional linking of a variety of motor cortical points, or rather the selection of fixed patterns embedded in the MCx circuitry. It will be suggested that both operational principles are probably involved. We also discuss the neural mechanisms by which cortical points may be dynamically linked to synthesize movement related muscle synergies. Separate corticospinal outputs sum linearly and lead to a blending of the movements evoked by activation of each point on its own. This operational principle may simplify the synthesis of motor commands. We will discuss two possible mechanisms that may explain linear summation of outputs. We have observed that the final posture of the arm when pointing to a given spatial location is relatively independent of its starting posture. From this observation and the recurrent nature of the MCx intrinsic connectivity we hypothesize that the basic mode of operation of the MCx is to associate spatial location to final arm posture. We explain how the recurrent network connectivity operates to generate the muscle activation patterns (synergies) required to move the arm and hold it in its final position.

## Introduction

What the motor cortex (MCx) does and how it does it are major scientific questions that remain unresolved. These issues are important because they are at the core of understanding cortical function. The MCx is, to paraphrase Sherrington, the final common cortical area where willful intention is translated into observable action. Its activation is the result of massive neural integration in a large number of cortical and subcortical areas (e.g., see Rizzolatti and Kalaska, 2013). Consequently, the MCx cannot be fully understood in isolation. Nonetheless, because of its vantage point, studying the MCx can further our understanding of what is being integrated and how. Here we propose that the MCx integrates kinematics and kinetics. Specifically, we hypothesize that the MCx associates the spatial location to which the limb is commanded to move with the respective muscle synergies required to move it and hold it in place, as required. Our hypothesis on this basic mode of operation of the MCx is developed in the final section of the article. On the way there we review and discuss several key issues concerning the functional organization of the motor output map, the nature of the connectivity between the different map loci, the mode of operation of the motor cortical circuitry and how they are all related.

## Topography of muscle representations in humans and animals

Mapping experiments based on electrical microstimulation of MCx in animals have demonstrated that a given muscle is represented at a multitude of non-contiguous loci and in various combinations with other muscles (e.g., Armstrong and Drew, 1985; Donoghue et al., 1992; Schneider et al., 2001). Schneider et al. (2001) showed unequivocally that such observations are not due to spread of stimulus current, or the result of conduction along intracortical axonal branches, to a single focus of representation (see also, Capaday, 2004). Subsequently, Rathelot and Strick (2006) used retrograde trans-neuronal transport of rabies virus injected in single digit muscles of macaques to study the distribution of corticospinal cells projecting to the respective motoneuron pool. This enabled them to identify cortico-motoneuronal (CM) cells that make monosynaptic connections with the motoneurons of the injected muscle. They found that the CM cells of a single digit muscles are spatially widespread and fill the entire mediolateral extent of the arm area. Further, they emphasized that CM cells for digit muscles are found in regions of MCx that are known to contain the shoulder representation. The cortical territories occupied by CM cells for different muscles overlapped extensively. No evidence for a single focal representation of muscles in MCx was found. They concluded that the “*overlap and intermingling among the different populations of CM cells may be the neural substrate to create a wide variety of muscle synergies*,” as had been previously demonstrated (Schneider et al., 2001) and emphasized (Phillips, 1975; Capaday, 2004).

**Figure 1:**
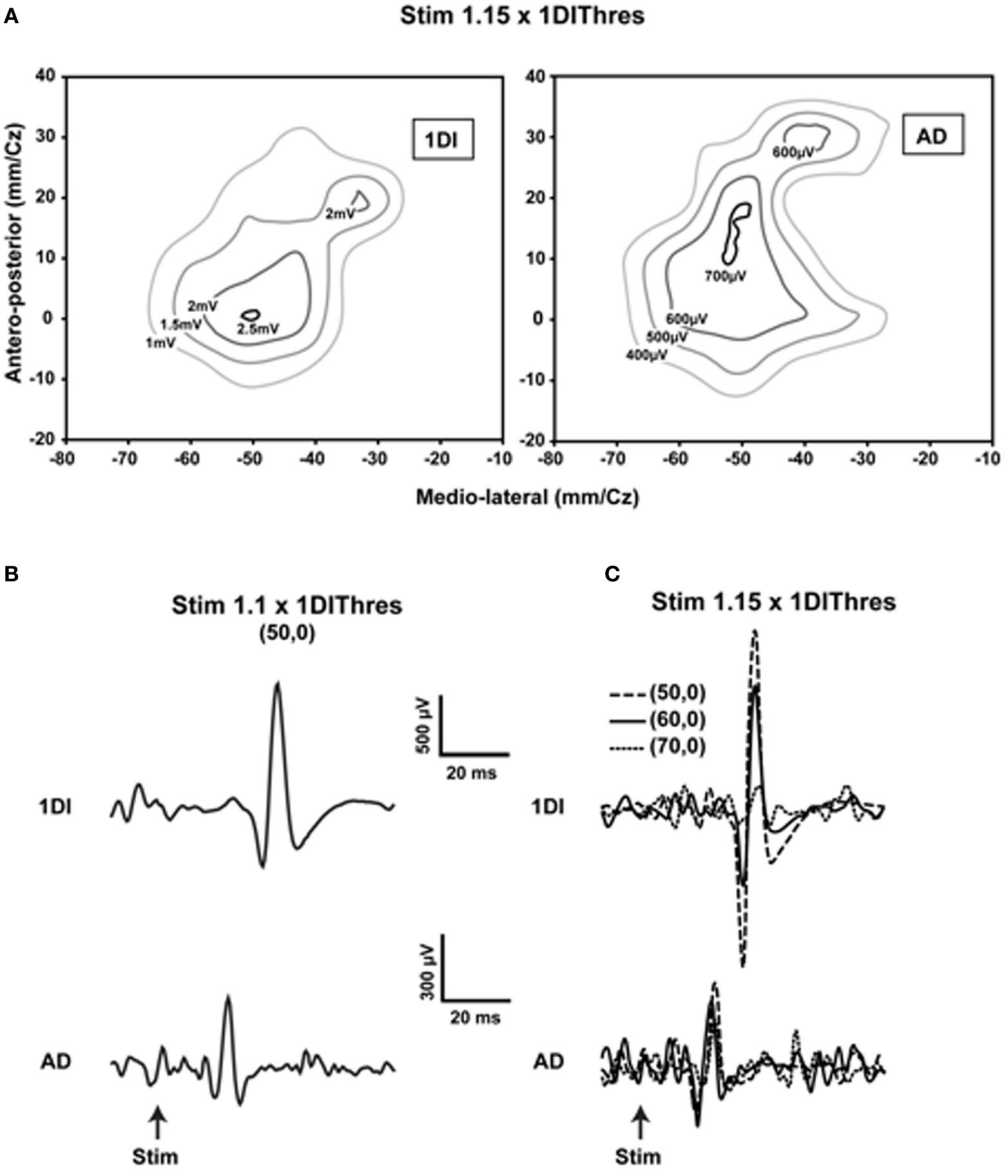
**Evidence showing that current spread does not explain the overlap of representations. (A)** Contour plots of the first dorsal interosseus (1DI) and anterior deltoid (AD) in a single subject obtained at 1.15 times the active motor (AMT) threshold of the 1DI. Note the slightly larger representation of the AD and the considerable overlap of the two representations. Note also that the optimal points are within 10 mm of each other in the antero-posterior direction and essentially coincident in the medio-lateral direction. **(B)** When the stimulus is reduced to 1.1 × AMT of the of the 1DI and the stimulus applied at the 1DI optimal point, MEPs are elicited in both the 1DI and AD. Note that in this case the stimulus is at the AD threshold. **(C)** Movement of the coil laterally in steps of 10 mm, starting at coordinate (50, 0), reduces the 1DI MEPs significantly, whereas the AD MEPs are relatively more constant despite the fact that the coil was moved further away from its optimal point than it was from that of the 1DI. This demonstrates that the measured overlap of the AD and 1DI representations is not due to current spread [reproduced with permission from Devanne et al. (2006)].

Is the human MCx similarly organized? A detailed mapping study using transcranial magnetic stimulation (TMS) showed the essential likeness of human and animal motor cortical maps (Devanne et al., 2006). They found that areal representations of commonly used proximal and distal muscles overlap considerably, despite differences in the location of their optimal points. What was new in that study was their demonstration that, as with the animal studies, the observed overlap was not due to current spread (Figure 1). Furthermore and contrary to often encountered descriptions of human motor cortical maps, the areal representations of commonly used proximal and distal muscles—anterior deltoid (AD), extensor carpi radialis (ECR), and first dorsal interosseus (1DI)—are similar in size. The comparable areal representation of the single muscles AD, ECR, and 1DI does not imply, however, that the total areal representation of the shoulder, wrist and hand are of similar size. There are about 22 muscles in the arm; nine muscles move the shoulder and five the wrist (Alexander, 1992). By contrast, about 29 intrinsic and extrinsic muscles move the hand (Alexander, 1992). It is therefore not surprising that the hand area may occupy a larger motor cortical territory than that of the shoulder or wrist (Penfield and Rasmussen, 1950). What the results of Devanne et al. (2006) demonstrate is that commonly used shoulder, wrist and intrinsic hand muscles, taken singly, are represented in areas of similar size. The relatively large AD representation seems relevant to explaining the accuracy of human pointing and reaching movements (Lacquaniti and Soechting, 1982). An angular positioning error at the shoulder leads to a larger error between hand and target than a comparable angular positioning error at an index finger joint. The large representation of the AD would suggest that the finesse of motor cortical control of the AD may be comparable to that of finger muscles. More importantly, the shoulder is involved either as a base of postural support for movements of the forearm and hand, or in their transport. The large representation of the AD and its overlap with forearm and hand muscles is a likely neural substrate of such motor coordinations. Perusal of simian motor cortical maps obtained by microstimulation shows a large number of zones in which wrist, elbow, and shoulder representations are intermingled (e.g., Gould et al., 1986; Donoghue et al., 1992). The number of motor cortical sites from which shoulder muscles were activated was nearly equal to those from which wrist muscles were activated (Donoghue et al., 1992). Park et al. (2001) demonstrated in rhesus monkeys a specific motor cortical region containing neurons that represent functional synergies of distal and proximal muscles. The results obtained in human subjects are in fact rather similar to those obtained in animals. Despite considerable overlap of representations found in the human MCx by Devanne et al. (2006) and others (e.g., Wassermann et al., 1992; Krings et al., 1998), the optimal point of the AD is on average more medially situated along the motor strip than those of the more distal muscles ECR and 1DI. Thus, the classic notion that proximal muscles are represented more medially along the motor strip than distal muscles is not without merit, but the overlap of representations must be emphasized. It is also important to consider that experiments using spike-triggered averaging of rectified EMG activity in monkeys (McKiernan et al., 1998) showed that over 45% of recorded CM cells facilitated at least one proximal muscle (elbow or shoulder) and at least one distal muscle (wrist, digit, and intrinsic hand muscles). On the assumption that this is also the case in humans, it is difficult to see how discrete non-overlapping representations can be obtained.

**Figure 2:**
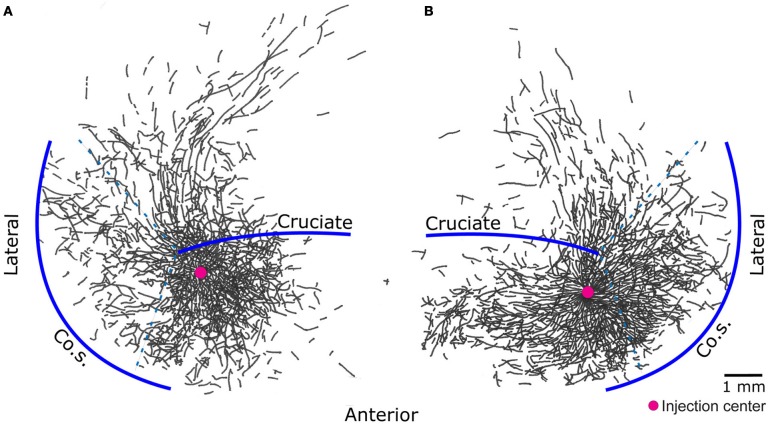
**Pyramidal cells of the cat motor cortex give rise to an extensive network of horizontal axon collaterals.** The motor cortex is bounded laterally by the coronal sulcus (Co.S.), the location of the coronal gyrus is demarcated by the dashed lines - - -. The areas anterior and posterior to the cruciate sulcus, along with the coronal gyrus, constitute the cat primary motor cortex. Biocytin, an anterograde tracer, was injected in points marked by a red circle. Threshold level microstimulation at that point evoked activity in the brachialis muscle in **(A)** and the extensor carpi radialis in **(B)**. Note how the axon collaterals of pyramidal cells from both deep and superficial layers of a small cortical locus spread to cover nearly all of the forelimb representation area [figure modified from Capaday et al. (2009)].

The results presented here are consistent with the Jackson–Walshe perspective on the functional organization of the MCx, viz. that the MCx represents complex patterns of overlapping and graded movement/muscle representations (see Walshe, 1943; Capaday, 2004, for a historical account and Phillips, 1975, for a discussion doing away with the muscles vs. movements controversy). The muscles of the arm are not controlled singly and separately, a point that was made right at the genesis of research on the MCx (Jackson, 1882^1^). For one, individuated control does not make sense biomechanically, as torques generated at one joint produce motion at other joints. Additionally, a large number of muscles cross more than one joint and thus produce movements at all spanned joints (e.g., the effect of long finger flexors on the wrist). While individuated movements at single joints are possible, they do not represent the plurality of movements ordinarily executed. Such movements often involve activation of multiple muscles to stabilize other joints so as to counteract actions of multi-joint muscles and segmental interaction torques. Furthermore, this ability does not imply that the MCx controls the musculature singly and separately, as will be discussed further on. The intermingled and re-duplicated muscle representation pattern is consistent with and provides a basis for the idea that the upper limb is controlled in an integrated manner (Capaday, 2004). Still, taken by itself, this organizational feature can be interpreted as a piano keyboard type of arrangement (see Graziano, 2006, for a historical account). However, cortical points are not isolated from each other, they are interconnected by long range intrinsic collaterals (Huntley and Jones, 1991; Keller, 1993; Lund et al., 1993; Capaday et al., 2009). Thus, in the cat, no two cortical points are fully independent, over distances spanning up to 6–7 mm (Figure 2). The nature of this connectivity and its implications for the mode of operation of the MCx are considered next.

## On the nature of the intrinsic connectivity of the MCx

**Figure 3:**
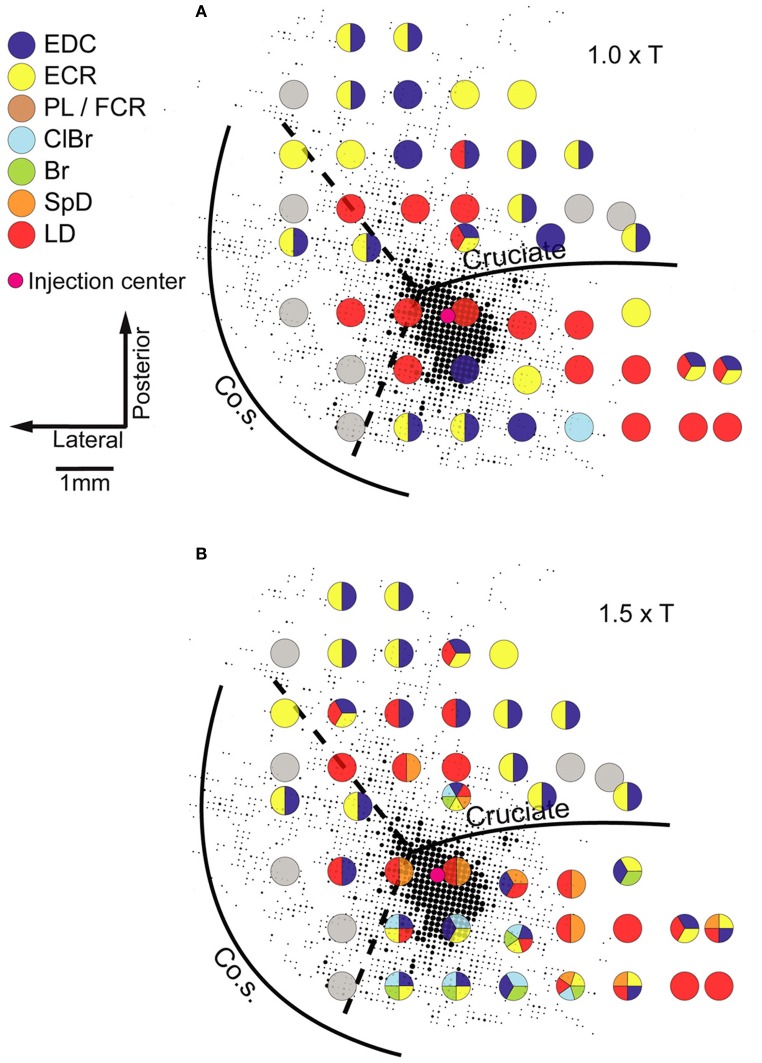
**Example of a superposition of the bouton density map on the microstimulation derived muscle map obtained in the same animal.** The size of each dot is proportional to the number of boutons in a grid element of 83 × 83 mm, quantitative details may be found in Capaday et al. (2009). Evoked muscle responses at each point at which microstimulation was applied are represented by the color code of the legend (left). Gray circles represent points at which no response was obtained (NR). Note that at many cortical points more than one muscle was recruited. **(A)** The microstimulation derived muscle map at 1.0× threshold. **(B)** The muscle map obtained at 1.5× threshold. Muscle abbreviations: EDC, extensor digitorium communis; ECR, extensor carpi radialis; PL, palmarus longus; FDP, flexor digitorium profundus; ClBr, clavo brachialis; Br, brachialis; SpD, spino deltoid; LD, latissimus dorsi. Reproduced from Capaday et al. (2009).

Neuroanatomical studies in monkeys and cats have unambiguously demonstrated strong intrinsic connectivity between widespread areas of the MCx (Huntley and Jones, 1991; Keller, 1993; Lund et al., 1993; Capaday et al., 1998, 2009). Indeed, numerous electrophysiological studies have demonstrated lateral interactions between neurons of the MCx (Matsumura et al., 1996; Baker et al., 2001; Jackson et al., 2003; Smith and Fetz, 2009). Here we limit the discussion to the anatomical aspects. An example of the widespread intracortical connectivity of the cat MCx is shown in Figure 2. The axon collaterals are studded with synaptic boutons all along their course (Capaday et al., 2009), which may be inferred from their beaded appearance in Figure 2. The intrinsic connections of a cortical area outnumber its feedforward (inputs) and feedback (top-down) connections (e.g., White, 1989; Dayan and Abbott, 2001). Understanding the function of intrinsic connections is therefore fundamental to understanding the neural processing that occurs within a cortical area. Predicated on this idea, Capaday et al. (2009) linked anatomy and physiology in finer detail than previous studies. Motor output was measured by intramuscular EMG recordings from up to 10 muscles making for a detailed output map. Axonal collaterals were traced from origin to termination with special care to identify the synaptic boutons along their course using correlative light and electron microscopy. Superposition of the synaptic bouton distribution map and the motor output map revealed that motor cortical neurons do not make point-to-point connections, but rather bind together the representations of a variety of muscles within a large neighborhood (Figure 3). Spiking activity at a cortical point may thus potentially influence any other cortical point within its innervation territory. This would allow for synergistic interactions between arbitrary cortical points giving rise to a rich repertoire of possible movements. The Jackson–Walshe perspective of overlapping and graded movement representations finds credence in the relation between the intrinsic connectivity and motor output maps.

Two other features of the maps shown in Figure 3 stand out. The dense core of bouton connectivity surrounding the injection point and the obvious intermingling of muscle representations. The dense core of connectivity has an area of about 3 mm^2^. Note also in Figure 3 that as the stimulus intensity is increased, responses from more muscles may appear and that their identity is not readily predictable from the responses of nearby points obtained at lower intensity. Such observations strongly argue against the idea that stimulus spread explains the recruitment of additional muscles with increasing stimulus strength. The more sensible conclusion is that the activation thresholds are different for the varied muscles that may be represented at a given cortical point. Capaday et al. (2009) also reported that excitatory and inhibitory neurons in the innervation territory of a cortical point receive synaptic inputs. This is nicely consistent with White's (1989) first canonical cortical circuit principle, viz. that “*every neuron in the target region of a projection receives input from the projection*” and, importantly, its corollary which states that “*axon terminals from any extrinsic or intrinsic source synapse onto every morphological or physiological neuronal type within their terminal projection field* … ” This feature of the cortical circuitry is consistent with a balanced neural network as proposed by Van Vreeswijk and Sompolinsky (1996). A key property of balanced networks is that the population output is a linear function of the input, despite non-linear unit properties. We will take up this issue again in section “A Hypothesis on the Basic Mode of Operation of the MCx” when discussing the neural mechanisms underlying the linear summation of MCx outputs and their functional significance. The third feature that may be inferred from the connectivity pattern is its recurrent nature. Cortical points, within the limits of axon collateral lengths, are reciprocally connected. Recurrent networks have a property which appears relevant to MCx function as we see it, they can function as hetero-associative systems (e.g., see Dayan and Abbott, 2001; Trappenberg, 2002). Our hypothesis on the basic mode of operation of the MCx is that it associates the spatial location to which the limb is commanded to move with the respective muscle synergies required to move it there and hold it in place, as required. The details will be presented in the final section of this article.

**Figure 4:**
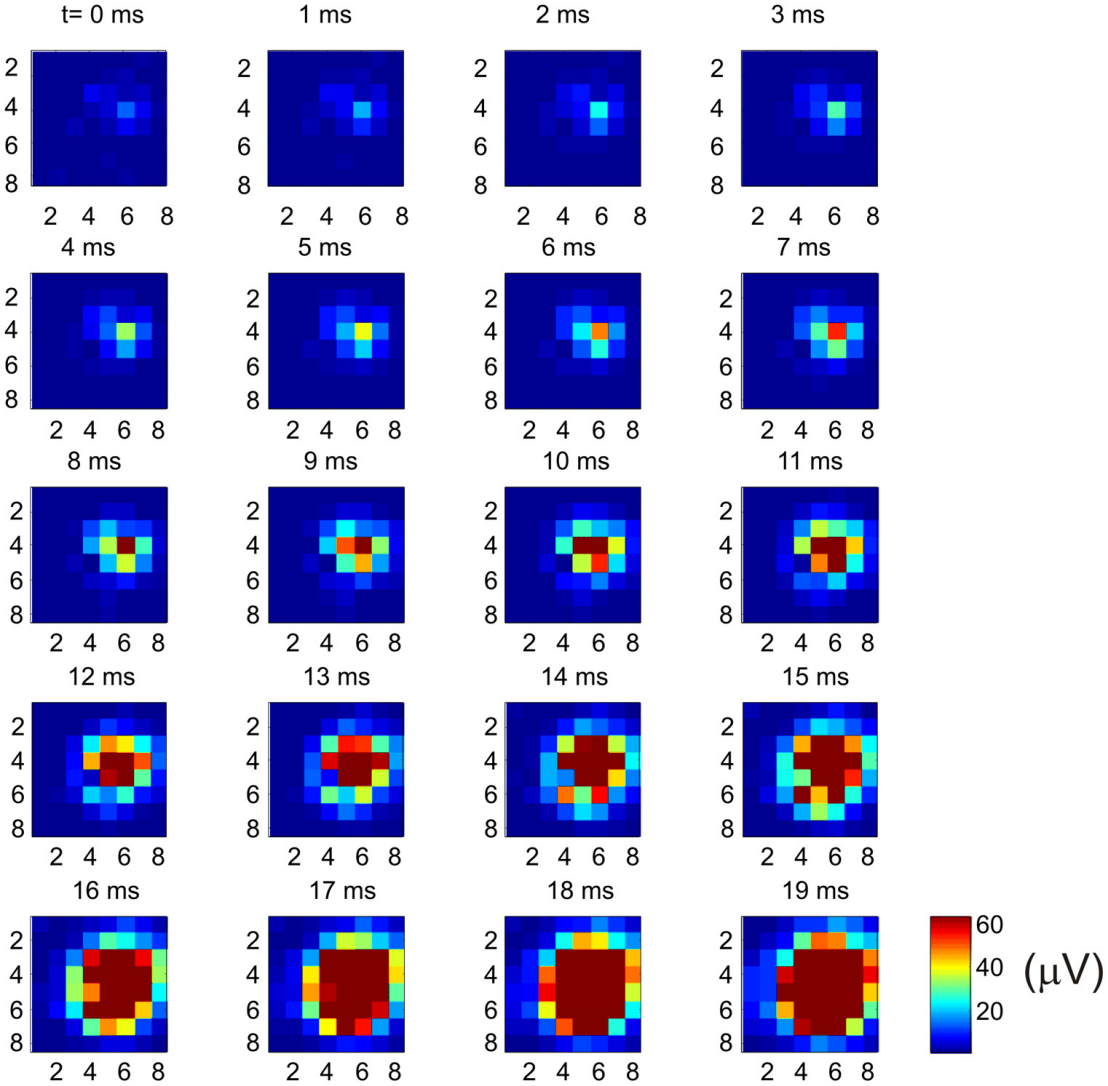
**Example of how multi-unit activity (MUA) recorded by an 8 × 8 Utah array propagates from a cortical point.** Bursting spike activity in the MCx was produced by focal iontophoretic ejection at array coordinate (6, 4) of the GABA_A_ receptor antagonist Bicuculline. The maps of neural activity were calculated every millisecond from near the onset (*t* = 0 ms) of spontaneous bursts of ictal neural activity to the time at which the maximum cortical area was recruited (*t* = 19 ms). Activity continues for several tens of millisecond after that. Note the onset of activity at coordinate (6, 4) and the subsequent progressive recruitment of cortical territory with time. In this example activity was evoked at 62 out of 64 possible electrodes. The recruited cortical area was 7.6 mm^2^, or 96% of the area covered by the array [reproduced with permission from Capaday et al. (2011)].

Why does the recurrent network pattern of the intracortical connectivity change the picture of how the MCx may function? The answer, as we have already stated, is that activity at a cortical point can spread and activate nearby cortical points where different muscle groups are represented. But how far does neural activity actually spread? In the cat MCx, neural activity generated at a cortical point about 400 mm in radius spreads at a velocity of 0.1–0.24 m/s to recruit a cortical area of some 7.22 mm^2^ (Capaday et al., 2011). The physiologically recruited cortical area is smaller than the area covered by the anatomical connections, but larger than the dense core of connectivity (Figure 4). From the aforesaid, we can infer that neural activity spreads over a radial distance of about 1.5 mm, when the balance between synaptic excitation and inhibition is not upset. However, cortical points up to 6–7 mm apart can be functionally coupled by reducing the strength of inhibition at one of the points (Schneider et al., 2002). This observation has led to the suggestion that motor commands may be synthesized by coupling cortical points through selected excitation and release of inhibition (Schneider et al., 2002; Capaday, 2004). It is clear therefore that an input to MCx will activate a cortical area whose size will depend on the intensity of the input and the level of inhibition at cortical points with which it is connected. We can use the connectivity map to understand individuated movements, such as index finger flexion and extension, as well as the more common natural movements requiring coordination between joints, such as reaching for an object. Imagine that a small focalized input to MCx will produce motion at the index finger. But this is only possible if nearby articulations are stabilized. Clearly, the so called focalized activity is only part of the motor command structure. As the contraction strength is increased, it is a common observation that activity irradiates to other muscles. This is presumably due, at least in part, to the intracortical connectivity we have described. The irradiation of activity is not pathological, it is sensible as was understood by Hughlings-Jackson who wrote

“Because the movements of the thumb and fingers could scarcely be developed for any useful purpose without fixation of the wrist (and of parts further and further in automaticity according to the force required), we should a priori be sure that the centre discharged, although it might represent movements in which the thumb had the leading part, must represent also certain other movements of the forearm, upper arm, etc., which serve subordinately.”

(In Selected writings of Hughlings-Jackson, 1931, vol. 1, p. 69).

The intracortical connectivity may thus also be viewed as the structural basis of an anticipatory neural network, foreseeing what additional muscles may need to be recruited as the movement evolves, or is perturbed. In the case of coordinated multi-articular movements, a larger cortical territory is likely engaged. The size of the cortical area necessary to evoke such movements is not known, here we speculate that this may involve the area of dense core connectivity. But, if we also consider that the MCx is involved in commanding associated postural adjustments (Massion, 1992), the area is probably much larger. This may explain why various lines of investigation suggest that even for simple finger movements large areas of the MCx appear to be activated (e.g., Sanes et al., 1995; Devanne et al., 2002). In any case, as the input to a particular cortical point is increased and inhibition at surrounding points decreased, the intracortical connectivity insures the synergistic recruitment of muscles required to produce the movement. We do not understand in detailed mechanistic terms how the MCx controls movements, but the intracortical connectivity must be taken into account by any eventual theory. It will also be important for future studies to determine the source and nature of the inputs that initiate activity in the MCx. What seems clear in considering the topography of muscle representations and the intrinsic connectivity is that the MCx contains a large number of potential functional links between widespread muscles. How specific muscle synergies are selected by cortico-cortical and subcortical inputs during voluntary movements is a challenge for the future.

## Cortical control of antagonistic muscles

**Figure 5:**
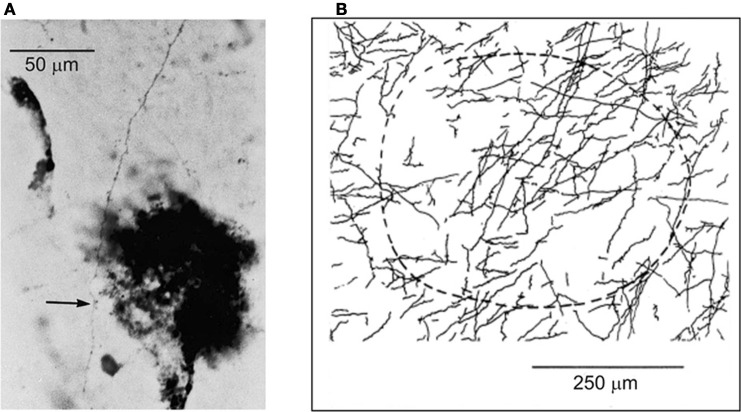
**(A)** Example of a biocytin stained axon collateral from a pyramidal neuron in a wrist extensor motor cortical point is shown coursing through a wrist flexor point identified by a small deposit of HRP (dark spot). The arrow points to an en-passant synaptic bouton on the axon collateral. **(B)** The camera lucida drawing of all axon collaterals coming from the wrist extensor point and coursing through the identified wrist flexor point. The dashed curve represents an area of approximately 250 mm in radius surrounding the center of the HRP deposit site.

Within the extensive intrinsic connectivity described in the preceding section, motor cortical points representing antagonistic muscles are also synaptically coupled by intrinsic axon collaterals (Capaday et al., 1998). In the example shown in Figure 5A biocytin was injected in a cortical point at which the ECR muscle (a physiological flexor) was represented. HRP was injected at another cortical point, about 2.8 mm away, at which its antagonist the palmaris longus muscle (a physiological extensor, or anti-gravity muscle) was represented. One can see a biocytin stained axon collateral studded with boutons along its course passing through a dense core of HRP staining. The camera lucida reconstruction of all the labeled collaterals coursing through the HRP deposit is shown in Figure 5B. The connections are excitatory, but they are normally held in check by local GABAergic inhibition, as we have shown in a subsequent physiological study (Ethier et al., 2007). Additionally, cortically mediated reciprocal inhibition operates at the spinal level when these points are activated, details of which will be discussed further on. Presumably, these interconnections are involved in the flexible control of antagonistic muscles, going from reciprocal activation to co-contraction. However, no studies of the function of this intra-cortical circuit have been done during behavior. Part of our message in this article is that to understand the MCx, is to understand how such circuits actually work during movement. By contrast to spinal circuitry, we are only at the beginning of relating cortical circuitry to motor function.

In our original study on the cortical control of antagonistic muscles (Capaday et al., 1998), as in all studies using microstimulation, cortical points that evoke a response in physiological extensor muscles (i.e., anti-gravity muscles) are few in comparison to those that evoke a response in physiological flexor muscles. Why might this be? There are two main reasons, as we discovered (Ethier et al., 2007). First, there is a strong asymmetry of cortically mediated reciprocal inhibition in the spinal cord. Cortically mediated inhibition is much stronger on physiological extensors than flexors (Ethier et al., 2007; see also references therein). This bias is particularly strong for wrist and elbow muscles, but less so for shoulder muscles (Ethier et al., 2007). This may be perhaps related to the functional role of the shoulder in providing a stable anchor for movements of the forelimb. Second, cortical points controlling antagonistic muscles are to a significant extent close together, or even commingled (Ethier et al., 2007). Consequently, the evoked descending volley is mixed; corticospinal fibers going to both flexor and extensor motoneuron pools are discharged. This volley will preferentially evoke a response in flexor motoneurons, because the cortically mediated reciprocal inhibition on the extensor motoneurons is strong. The same principle applies in humans (Capaday, 1997) when the MCx is activated by TMS. The asymmetry of the cortically mediated reciprocal inhibition strongly biases motor cortical maps derived by microstimulation, or TMS.

One should also be cautious of the simplified interpretations derived from such maps. As, for example, that the cat MCx excites forelimb physiological flexors and inhibits physiological extensors, or that by contrast in the baboon the converse is true (e.g., Preston et al., 1967). The latter account implies that for a baboon to reach for a food morsel the MCx controls the extension of the forelimb, but that the subsequent flexion movement to bring the morsel to its mouth would be mediated by a different part of the CNS. We suggest that a too literal interpretation of these otherwise sound data does not represent the true nature of motor cortical control. Preston et al. (1967) insightfully interpreted the strong cortical inhibition of physiological extensors in cats as part of a mechanism to arrest the tonic anti-gravity activity which occurs during standing postures. For the baboon, the interpretation was that it represented a neurophysiological sign of the transition from quadruped to biped posture. In neither case was it implied that motor cortical control has a unidirectional bias. Indeed, recent studies have shown that both types of movements can be elicited by microstimulation of the simian MCx (Graziano et al., 2002, 2004). Yet another factor that may bias cortical maps is the relative excitability of different motoneuron pools. Two factors are involved here; the input resistance of the motoneurons and spontaneous depolarizing drive that may occur in different states. Little is known about motoneuron input resistance differences between motor pools such as those of wrist and shoulder muscles. In principle, pools constituted of motoneurons having a higher input resistance will tend to be preferentially activated by synaptic currents.

## Feedback remapping of cortical outputs

Graziano et al. (2004) suggested the possibility that the output of cortical points may be remapped by proprioceptive inputs (see also Graziano, 2006). They demonstrated that, for example, microstimulation at a cortical point evoked either activity in the triceps muscle when the elbow was flexed, or activity in the biceps when the elbow is extended. In another example they showed that evoked activity in the triceps increased monotonically as a function of the degree of elbow flexion. In these as well as other examples, examination of the EMG recordings (e.g., Figure 9 in Graziano, 2006) shows that the evoked responses are a function of the background EMG activity in the respective muscle. When the elbow was flexed (triceps is stretched) the background activity increased in the triceps and its microstimulation evoked response also increased. When the elbow was extended (biceps stretched) the background EMG activity of the biceps increased and so too its evoked response. We propose that this can be explained by changes in spinal neural circuit excitability produced by the stretch reflex, the associated reciprocal inhibition and the close grouping or intermingling of the corticospinal neurons controlling the biceps and triceps, respectively. Thus, for example, when the biceps is stretched the increased spindle afferent discharges will increase the activity of the biceps motoneurons via the stretch reflex pathway(s) and reciprocally inhibits the triceps motoneurons. Consequently, the mixed corticospinal volley will evoke a net response in the biceps motoneurons. Graziano (2006) suggested that such observations are evidence for proprioceptive remapping of the output of cortical points by mechanisms intrinsic to the MCx and spinal cord. We suggest that these results depend only on spinal neural mechanisms, as explained. Relatedly, Griffin et al. (2011) have demonstrated that during ongoing voluntary motor activity high-frequency microstimulation of the MCx in macaques has effects which depend on the ongoing level of EMG activity, but not limb position, which can confound interpretation.

Nonetheless, how proprioceptive information is used by the motor cortical circuitry is an important issue that has not received much attention beyond establishing the existence of a trans-motor-cortical stretch reflex in primates, including humans (Phillips, 1969, 1975; Cheney and Fetz, 1984; Capaday et al., 1991). We will discuss the possible role of proprioception in the operations of motor cortical circuits in the final section of this article.

## Neural mechanisms of linear summation of MCx outputs

We do not know whether the MCx stores motor engrams (i.e., memories of complete movements) or, by contrast, whether it synthesizes a movement on a moment-to-moment basis by selecting multi-purpose muscle synergy modules and if so, how. One possibility, as discussed in the section “On the Nature of the Intrinsic Connectivity of the MCx,” is that the dense core of connectivity contains the neural circuitry, or engram, required to evoke a movement. However, as the horizontal connections extend beyond the dense core, it may be possible to functionally link cortical points representing different muscles, or muscle synergies. Such a mechanism would allow creating a rich variety of movements, from a smaller repertoire of stored basic engrams. We discussed in section “On the Nature of the Intrinsic Connectivity of the MCx,” how selected excitation and release from inhibition can functionally link distinct cortical points and produce a synergistic motor output pattern (Schneider et al., 2002). Whatever the mechanisms of muscle synergy selection turn out to be, it seems important to understand quantitatively how cortical points interact and how the net output is thereby modified.

**Figure 6:**
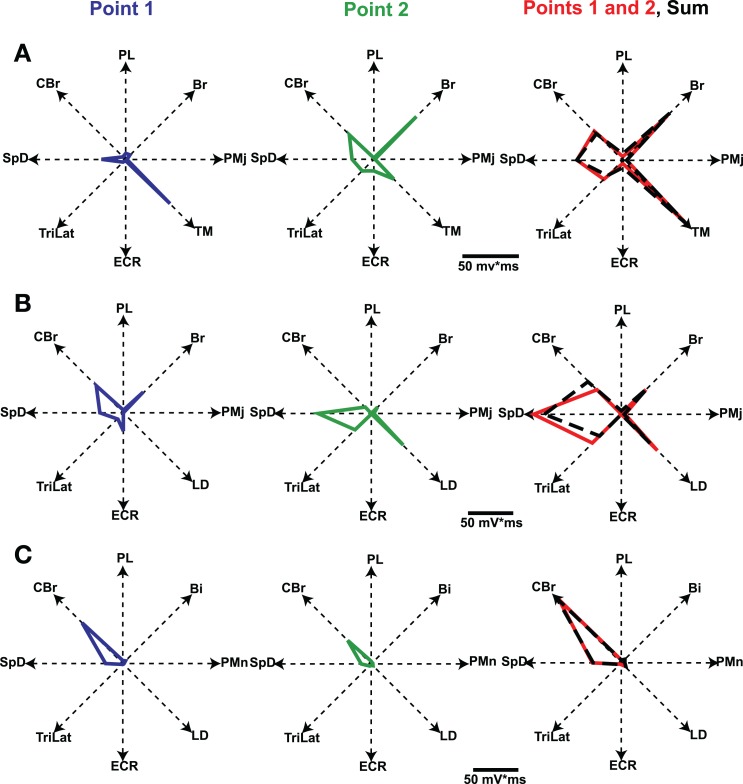
**Polar plots of evoked EMG activity and their summation.** Each axis represents the integrated EMG activity of a given muscle. Line segments join the points plotted on each axis, thus giving a geometrical representation of the evoked muscle-coordination pattern. Graphs in the first two columns represent the muscle-coordination pattern evoked by separate stimulation of two cortical points. Graphs in the third column represent the muscle coordination pattern obtained when the two points were simultaneously stimulated (Points 1 and 2, red line) and the linear sum expected by addition of the two separate patterns (black dashed lines). Note that the expected and experimentally obtained muscle-coordination patterns are nearly the same in the three examples shown **(A–C)**. Figure from Ethier et al. (2006).

In the study by Ethier et al. (2006) we asked how the outputs of two simultaneously stimulated motor cortical points summate. To this end experiments were done in Ketamine anesthetized cats. Long trains (e.g., 500 ms) of intracortical microstimulation applied to the MCx evoked coordinated movements of the contralateral forelimb, as was first shown by Graziano et al. (2002) in the monkey. Paw kinematics in three dimensions and the EMG activity of eight muscles were simultaneously recorded. The evoked movements were represented as displacement vectors pointing from initial to final paw position. We showed that the EMG outputs of two cortical points simultaneously stimulated sum linearly (Figure 6). Additionally, the displacement vector resulting from simultaneous stimulation pointed in nearly the same direction as the algebraic resultant vector. This result is true as long as the individual movement vectors point in different directions and are not due to motion at single joint, which rarely if ever occurs with long duration trains of microstimulation. Importantly, however, the resulting movement during simultaneous stimulation is always a blend of the movements evoked from each cortical point on its own (Ethier et al., 2006). Linear summation of EMG outputs was also found when inhibition at one of the cortical points was reduced by GABA_A_ receptor antagonists. A simple principle emerges from these results. MCx outputs combine nearly linearly in terms of muscle activation patterns, despite the underlying complex neuronal circuitry and electrophysiological properties of neurons. The summation of muscle activation patterns leads to a blending of the movements evoked from each point. This operational principle may simplify the synthesis of motor commands, as previously discussed. Nonetheless, the linear summation of outputs was unexpected and puzzling. It is even more puzzling given the lack of effect of reducing inhibition at one of the cortical points; a condition in which it should have received the full brunt of inputs from the other cortical point.

There are at least two explanations for the observed linear summation of MCx outputs. The simplest is that the distance between paired cortical points was on average greater than that over which neural activity spreads, which is approximately 1.5 mm in radial distance as discussed in the section “On the Nature of the Intrinsic Connectivity of the MCx.” The distances between pairs of microstimulated points ranged between 0.66 and 5.7 mm, with a mean distance of 2.65 mm (*SD* = 1.52 mm). Thus, the separation between cortical points studied by Ethier et al. (2006) was on average greater than that over which activity at a cortical point influences its surround. It is possible, therefore, that we were dealing with effectively functionally isolated cortical points. However, the observation that despite reducing the strength of inhibition at one of the cortical points the outputs still summed linearly is more difficult to explain on these grounds. The distances between pairs of points tested in this way was between 2.65 and 4.62 mm, with a mean distance of 3.4 mm (*SD* = 0.92 mm). Thus, these pairs of cortical points were well within the range over which they can be functionally coupled, i.e., at least 5 mm (Schneider et al., 2002). Yet, despite the fact that in this condition spiking activity initiated at the stimulated point produces spiking activity at the disinhibited point, the outputs still summed linearly. This raises a second and more interesting possibility that the motor cortical circuitry may be wired to produce linear interactions between loci. Balanced neural networks as originally proposed by Van Vreeswijk and Sompolinsky (1996) involve a feedback dependent balance between excitation and inhibition such that, despite non-linear unit properties, the population output is a linear function of the input to the network. More recently, Capaday and Van Vreeswijk (2006) proposed a mechanism by which gain may be modulated by such a balance of excitatory and inhibitory synaptic inputs on dendritic trees. This may allow for the scaling of motor commands.

**Figure 7:**
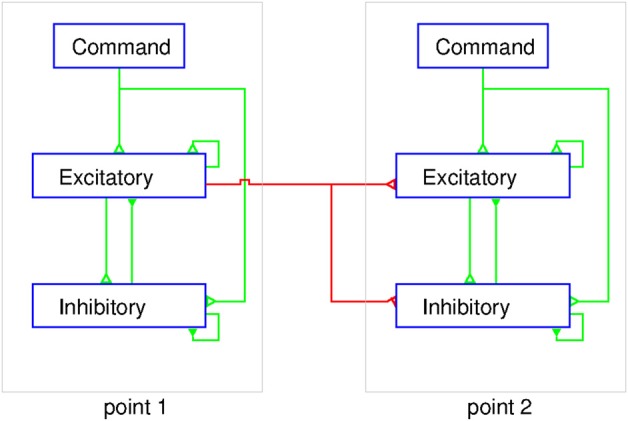
**Schematic representation of the balanced network model of MCx.** At each point the excitatory and inhibitory populations of neurons are interconnected and receive a command input from outside the MCx. The excitatory neurons also project to both the excitatory and inhibitory neurons in other cortical points. The figure shows two cortical points and for simplicity only the connections from point 1 to point 2 are shown (red lines). The corticospinal axons would emanate from a sub-group of the excitatory neurons at each point. The external command input makes synaptic contact with excitatory and inhibitory neurons simultaneously. The coupling within the intra-motor-cortical network neurons leads to a balanced state and linear summation of the separate outputs, as explained in the text.

In a balanced neural network (Figure 7) the sum of the excitatory currents from external inputs, as well as from the activity of intrinsic circuit neurons, is balanced nearly exactly by the recurrent inhibitory currents. Spiking occurs at times when noise fluctuations exceed threshold, thereby also explaining spike time variability. The basic idea of the balanced neural network is not unlike the principle used in operational amplifiers, where negative feedback of a portion of the output results in a device with linear input/output properties. Now consider a network consisting of multiple cortical points. The excitatory and inhibitory neuron populations at each point mutually interact and can receive external command inputs (Figure 7). The excitatory neurons at one point also projects to excitatory and inhibitory neurons at other cortical points through long range collaterals. If we neglect the latter for a moment, a command input into a single point would activate the excitatory and inhibitory cells and their activity evolves to a state where, in both populations, the inhibitory feedback roughly cancels the command input and the recurrent excitation. It can be shown that this results in a response in both populations of neurons which is proportional to the command input (Van Vreeswijk and Sompolinsky, 1996, 1998). When considering interacting cortical points, the problem is more complex. A command input into point 1 activates both neuron populations at that point. Through the horizontal connections, however, this produces an input in other points. This will activate the inhibitory cells in these points, and as a result, the excitatory cells receive excitatory inputs through the horizontal connections and local recurrent inhibitory inputs, as shown in Figure 7. If the ratio of the strength of synaptic inputs coming from the horizontal connections to the excitatory and inhibitory populations at a given cortical point is just right, this input to the excitatory cells is just canceled by the local inhibitory feedback. Thus, even though cortical points are connected, activation of one point may not recruit the excitatory cells at other points.

How does this explain the twin stimulation experiments? When point 1 is stimulated, it leaves the excitatory cells at point 2 unaffected, and *vice versa* (Figure 7). When both points are stimulated simultaneously, each point reacts to the stimulus input and the input from the other point. Since, in the balanced network, the response is linear with the external input, activity of both populations of neurons at each point is just the sum of the activity due to the stimulation and that due to input from the other point. But, since the latter does not affect the activity of the excitatory population, stimulation of one point does not affect the response of the excitatory population at the other point. As a result, the total output from MCx to the motoneurons in the spinal cord is the sum of the outputs to stimulation of these points separately. This model suggests that the long range connections have been carefully arranged to have no effect. This immediately raises the question of why they exist? We suggest that they serve to couple cortical points as needed for movement production, an idea proposed initially by Schneider et al. (2002). The lack of effect of activity at point 1 on point 2 is due the local inhibitory feedback at that point and vice versa (Figure 7). However, if the local inhibitory feedback is modified, for example through disinhibition, the anatomical connections between cortical points can be made physiologically relevant, i.e., cortical points can be functionally coupled. Thus, for example, proprioceptive inputs could by inhibiting inhibitory neurons at a given cortical point, allow it to respond when the command signal arrives.

The anatomical and physiological data are, in broad terms, in agreement with the theory of balanced networks. As discussed in the section “On the Nature of the Intrinsic Connectivity of the MCx,” intrinsic and extrinsic inputs to a cortical locus contact local excitatory and inhibitory neurons and the connections between these neurons are recurrent (i.e., there is feedback between them). This suggests that cortical neurons are driven by simultaneous excitatory and inhibitory currents, an idea consistent with recent physiological results (Haider et al., 2006; Okun and Lampl, 2008). The spiking activity of cortical neurons is thus not due to excitatory synaptic inputs alone, but rather the result of simultaneous excitation and inhibition. However, whether balanced network operations in the MCx explain why the output of cortical points sum linearly will require further experimental and theoretical investigations. In particular, the experimental data does not allow us at this time to understand the effects of disinhibition quantitatively. One possibility is that, when the second point is strongly disinhibited it enters into a limit cycle in which the strong local excitatory feedback leads to recurrent bursting activity. The input from point 1 via the horizontal connections is then presumably relatively small in comparison to the local inputs and would only serve to reset the phase of the limit cycle. Consequently, the total input to the motor pool would be the output due to stimulation of point 1 added linearly to the output from the spontaneous activity of point 2, as experimentally observed (Ethier et al., 2006). We are currently re-examining this issue.

**Figure 8:**
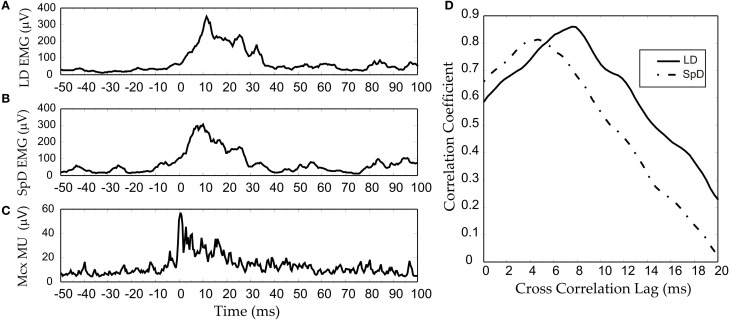
**An example of an RMS-smoothed multi-unit (MU) burst (C) recorded recorded from the cat MCx and simultaneously recorded EMG activity (A,B) of the two muscles in which activity was evoked. (D)** The cross-correlation function between the MCx waveform and the respective EMG waveforms. Bursting spike activity in the MCx was produced by focal iontophoretic ejection at a cortical point of the GABA_A_ receptor antagonist Bicuculline (see Capaday et al., 2011). The EMG recording where low-pass filtered at 100 Hz, whilst the MCx spiking activity was filtered at 1 KHz. The waveforms are averages of eight consecutive responses.

The two explanations we have proposed depend, nonetheless, on linear corticospinal transmission. It may also be conjectured that any non-linearity at the cortical level is compensated by a non-linearity of opposite direction at the corticospinal level. However, the results we have obtained from multi-unit-activity (MUA) recordings make this unlikely (Capaday et al., 2011). MUA recordings represent the weighted average of single spike activity recorded within some 100 μm from the microelectrode tip (Buchwald et al., 1965; Buchwald and Grover, 1970; Legatt et al., 1980). Importantly, MUA recordings obtained from multiple cortical sites, when taken together, yielded more accurate predictions of movement parameters than any other intracortical signal (Stark and Abeles, 2007). Figure 8C shows an example of an averaged MUA burst from layer V neurons of the cat MCx. Recurrent multi-unit bursts were induced by iontophoretic ejection of Bicuculline, a GABA_A_ receptor antagonist, at a motor cortical point (Capaday et al., 2011). The figure also shows the averaged EMG activity of the two muscles in which activity was evoked by the cortical burst (Figures 8B,C). Note the similarity of all three waveforms. The cross-correlation function confirms the high degree of linear correlation between the MCx waveform and the respective EMG waveforms (Figure 8D). To a first order approximation, therefore, the corticospinal stage of synaptic transmission may be characterized as linear, or threshold-linear to be more precise (see also Townsend et al., 2006).

## A hypothesis on the basic mode of operation of the MCx

Ultimately we want to relate neural circuitry to function. However, without an understanding of the global function of the MCx such an undertaking will not be possible. In this final section we develop our current working hypothesis on the basic mode of operation of the MCx. By basic mode of operation, we mean the most elementary purpose for which it exists, which is to willfully move a limb from one position to another.

We hypothesize that the command inputs to the MCx are, whatever their origin, kinematic in nature. This is a base assumption, but it is consistent with a large body of evidence. Cortical areas having direct, or indirect, inputs to MCx encode spatial features such as, the location of visual and cutaneous stimuli in various reference frames, or the combined position of limb segments with respect to the body (e.g., see Rizzolatti and Kalaska, 2013). In the simplest case, we propose that the kinematic inputs specify where, for example, the arm must be moved to. The output of the MCx is related to muscle forces, those necessary to move the limb and those required for its postural support. We thus further hypothesize that a transformation from a kinematic to a kinetic (muscle) frame of reference occurs within the MCx. This is consistent with several studies (e.g., Ajemian et al., 2000; Trainin et al., 2007). We suggest that this does not occur in stages but automatically as a consequence of the projection of the external inputs onto the local connectivity. Consequently, unless one can manage to record from the axons of the input pathways, neural activity explicitly related to kinematic variables will not be experimentally observed. All MCx spike activities which can be recorded with present technology will be de facto related to muscle forces, that is coded in a muscle based reference frame, because the output layer V is strongly interconnected with the other cortical layers (Weiler et al., 2008). The essentially kinetic nature of MCx output is consistent with Evart's original finding that MCx neuron discharges are related to the muscular effort required to move inertial loads (Evarts, 1968), that under isometric conditions MCx neuron discharges are related to the exerted force (e.g., Smith et al., 1975) and that when the limb is free to move MCx neuron discharges are related to joint power (Scott et al., 2001), the product of force/torque and velocity. The linear correlation between MUA in MCx and EMG outputs shown in Figure 8 is fully consistent with these key studies. The penultimate element of our hypothesis is that the transformation of kinematic command input to a muscle output pattern is based on the recruitment of embedded muscle synergies, according to the various schemes we have discussed in previous sections. The recurrent connectivity of the MCx is such that it can function as an attractor neural network (e.g., see Dayan and Abbott, 2001; Trappenberg, 2002). The input command to MCx will produce transient neural activity that, because of the recurrent connectivity, will settle to a steady-state activity pattern, the attractor state. In the process, this neural activity generates the required components of a motor command. A transient component that will drive the limb to the desired position and a steady-state, or tonic, component that will hold the limb in place. The transient component may recruit different muscles than those recruited by the tonic component. For example one may point to a location along the body's midline, but the arm may be initially located either to the left or to the right of that location. The muscle activities required to move the arm (transient component) is different in each case, but those required to hold the arm (tonic component) at that location are the same. The final element of our hypothesis is that the trajectory taken by the neural activity in the MCx as it settles to the steady-state depends on proprioceptive inputs to the MCx. This explains how different transient motor commands can be generated for the same kinematic input command.

**Figure 9:**
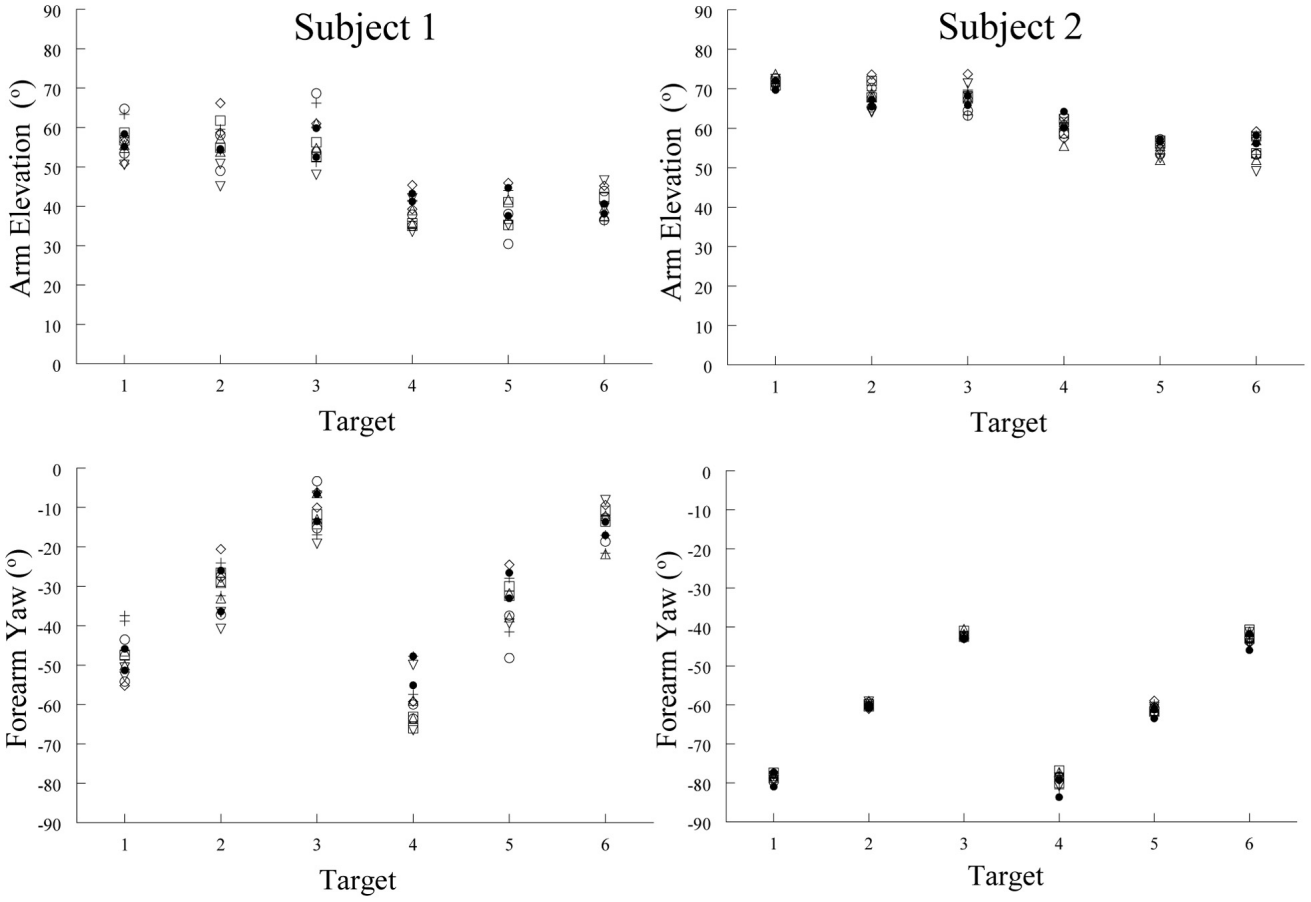
**Examples of arm elevation and forearm yaw angles characterizing arm posture at the end of pointing movements made to six different targets from seven different starting positions.** Note the low variability of these angular measures at each target locations. This demonstrates that arm posture at the end of a pointing movement to a given location was essentially the same, regardless of the starting position of the arm. Each symbol represents a movement made from one of the seven starting positions.

Our hypothesis implies that there should be some relation between spatial position and muscle activation pattern. Our study of the arm's posture at the end of pointing movements made by humans demonstrates such a relation. Limb posture is an indirect but accurate reflection of the muscle activation pattern, our tonic component, when the limb is held in place after a movement. We therefore measured the posture of the arm at six different locations (targets) in the workspace. The subjects were instructed to move the hand at a comfortable speed twice from each of seven widely spaced initial start positions to place the pad of the index tip slightly above the center of target cylinders. These were positioned at six different locations on a table-top in front of the subject. The arm elevation (angle of the humeral segment relative to its projection in the horizontal plane) and forearm yaw (angle of the forearm projected onto the horizontal plane) angles for the 14 movements to each target were similar. That is, the upper limb (arm and forearm) orientations were about the same for any one target location despite the varied start positions (Figure 9). As can be seen in Figure 9, the variability of the arm elevation and forearm yaw angles at each target location are relatively small and in fact independent of the initial start position. Put simply, regardless of where the arm is located before moving the fingertip to a given spatial location, the posture of the arm at that spatial location is relatively constant. This means that, by and large, Donder's law is obeyed for pointing movements of the arm. That is, a given location of the arm endpoint (index finger tip) is achieved with the same orientations of the upper limb joints. Our results and conclusion differ from those of Soechting et al. (1995). In their study, large variations of some 25–30 degrees in average angle of the vector perpendicular to the plane of arm were observed at four of the five target locations after movements from widely spaced starting positions. However, in their study the subjects were instructed to “*move their arm to touch the tip of the pointer*.” This leaves considerable freedom as to how to orient the fingertip relative to the pointer tip. In our task, there was no such ambiguity, as subjects were asked to place the pad of the index finger slightly over the center of the top of a short cylinder ~2.5 cm in diameter.

The observation that the posture of the human arm at a given spatial location is essentially the same regardless of the starting position is consistent with our hypothesis. The findings of Aflalo and Graziano (2006) are also accordant with our hypothesis. They showed that, in monkeys, the discharge of MCx neurons is significantly related to the posture attained by the arm at the end of freely made spontaneous movements. Furthermore, microstimulation of a cortical point evoked arm postures that matched the postures to which the neurons at that point were best tuned.

## Epilogue

In summary, it is the recurrent nature of the connectivity that makes it possible for a simple command input coded in a kinematic reference frame to set the MCx into action and automatically generate the transient and steady-state portions of the motor command. The computational scheme we propose would be difficult to implement in non-recurrent networks.

### Conflict of interest statement

The authors declare that the research was conducted in the absence of any commercial or financial relationships that could be construed as a potential conflict of interest.

## Footnotes

^1^Note the original cited references to Hughlings-Jackson may be found in the two volumes set “Selected writings” published in 1931. An excellent summary of his ideas on the organization and function of the motor cortex is in the paper titled “Some implications of dissolution of the nervous system” (“Selected writings” vol. II, p. 29).
